# PET-CT-guided *versus* CT-guided biopsy in suspected malignant pleural thickening: a randomised trial

**DOI:** 10.1183/13993003.01295-2023

**Published:** 2024-02-01

**Authors:** Duneesha de Fonseka, David T. Arnold, Helena J.M. Smartt, Lucy Culliford, Louise Stadon, Emma Tucker, Anna Morley, Natalie Zahan-Evans, Anna C. Bibby, Geraldine Lynch, Eleanor Mishra, Shahul Khan, Mohammed Haris, Henry Steer, Leon Lewis, Alina Ionescu, John Harvey, Kevin Blyth, Najib M. Rahman, Anthony E. Edey, Chris A. Rogers, Nick A. Maskell

**Affiliations:** 1 Academic Directorate of Respiratory Medicine, University of Sheffield, Sheffield, UK; 2 Academic Respiratory Unit, University of Bristol, Southmead Hospital, Bristol, UK; 3 North Bristol NHS Trust, Bristol, UK; 4 Bristol Trials Centre, Medical School, University of Bristol, Bristol, UK; 5 Royal Cornwall Hospital NHS Trust, Truro, UK; 6 University of East Anglia, Norwich, UK; 7 Norfolk and Norwich University Hospitals NHS Foundation Trust, Norwich, UK; 8 Royal Stoke University Hospital, Stoke, UK; 9 Gloucestershire Hospitals NHS Trust, Gloucester, UK; 10 Aneurin Bevan University Hospital Trust, Newport, UK; 11 University of Glasgow, Glasgow, UK; 12 Oxford NIHR Biomedical Research Centre, Oxford, UK; 13 Nuffield Department of Medicine, University of Oxford, Oxford, UK; 14 Chinese Academy of Medical Sciences Oxford Institute, Oxford, UK

## Abstract

**Background:**

Pleural biopsy is the gold standard for diagnosis of pleural malignancy but a significant proportion will have an inconclusive biopsy despite ongoing clinical suspicion of malignancy. We investigated whether positron emission tomography-computed tomography (PET-CT) targeted pleural biopsy is superior to standard CT-guided pleural biopsy following an initial non-diagnostic biopsy.

**Methods:**

The TARGET trial was a multicentre, parallel group randomised trial. Patients with a previous inconclusive pleural biopsy but an ongoing suspicion of pleural malignancy were randomised (1:1) to receive either CT-guided biopsy (standard care) or PET-CT followed by a targeted CT biopsy (intervention). The primary outcome was pleural malignancy correctly identified from the trial biopsy.

**Results:**

Between September 2015 and September 2018, 59 participants were randomised from eight UK hospital sites: 29 to CT-only followed by targeted biopsy and 30 to PET-CT followed by targeted biopsy. The proportion of pleural malignancy correctly identified was similar between the groups (risk ratio 1.03 (95% CI 0.83–1.29); p=0.77). The sensitivity of the trial biopsy to identify pleural malignancy was 79% (95% CI 54–94%) in the CT-only group *versus* 81% (95% CI 54–96%) in the PET-CT group.

**Conclusions:**

The results do not support the practice of PET-CT to guide pleural biopsies in patients with a previous non-diagnostic biopsy. The diagnostic sensitivity in the CT-only group was higher than anticipated and supports the practice of repeating a CT-guided biopsy following an inconclusive result if clinical suspicion of malignancy persists.

## Extract

Pleural malignancy represents either metastases from another primary site (often lung, breast or ovarian) or primary pleural malignancy from pleural mesothelioma [1]. Pleural malignancy, especially pleural mesothelioma, can be challenging to diagnose due to the patchy distribution of heterogenous tumour across the pleural surface [2]. Pleural biopsy *via* image guidance or thoracoscopy has a false negative rate of 10–25% [3, 4]. For those with a non-diagnostic first pleural biopsy, but ongoing clinical or radiological suspicion of malignancy, the optimal approach is unclear.

## Tweetable abstract

**The TARGET trial was a multicentre randomised trial comparing PET-CT targeted pleural biopsy to standard CT-guided pleural biopsy for suspected pleural malignancy. The results do not support the practice of PET-CT to guide pleural biopsies.** https://bit.ly/40HOY6p

## Graphical abstract

**Figure:**
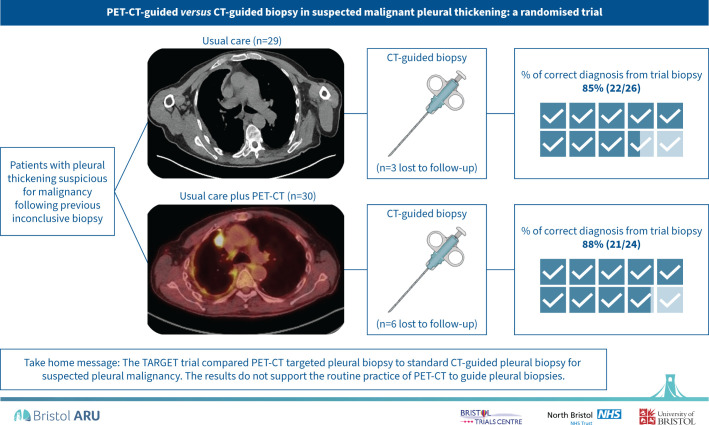
Summary of the TARGET trial. PET: positron emission tomography; CT: computed tomography.

## Introduction

Pleural malignancy represents either metastases from another primary site (often lung, breast or ovarian) or primary pleural malignancy from pleural mesothelioma [1]. Pleural malignancy, especially pleural mesothelioma, can be challenging to diagnose due to the patchy distribution of heterogenous tumour across the pleural surface [2]. Pleural biopsy *via* image guidance or thoracoscopy has a false negative rate of 10–25% [3, 4]. For those with a non-diagnostic first pleural biopsy, but ongoing clinical or radiological suspicion of malignancy, the optimal approach is unclear.

Positron emission tomography-computed tomography (PET-CT) has been shown to be highly effective in the diagnostic pathway of other malignancies, including lung cancer, to both fully stage and guide optimal biopsy sites. The role of PET-CT in suspected pleural malignancy is less well defined. Theoretically, the radioactive tracer material 18-fluorodeoxyglucose is taken up by highly metabolically active tissue, such as neoplastic tissue [5]. These areas can then be biopsied under image guidance for a more accurately targeted biopsy of a metabolically active area. However, pleural malignancy is a very heterogenous disease and the actual role of PET-CT for the indication of targeting pleural biopsies in those with one non-diagnostic biopsy is not known.

In this randomised controlled trial (RCT), we aimed to investigate whether addition of PET-CT to target pleural biopsy is superior to a standard CT-guided pleural biopsy in patients with ongoing pleural thickening suspicious for malignancy, following a first non-diagnostic biopsy.

## Methods

### Trial design

The TARGET trial was a multicentre, parallel group RCT. Following written informed consent, participants who had a previous inconclusive pleural biopsy were allocated in a 1:1 ratio to receive either standard care (CT-guided biopsy only) or the intervention (PET-CT followed by a targeted pleural CT-guided biopsy). Study participants were followed up for up to 12 months after randomisation. Ethical approval was granted by the South-West Research Ethics Committee (15/SW/0156). The trial was sponsored by the North Bristol NHS Trust. The trial protocol has been published previously [6]. There was a short delay in the registration of the trial on the ISRCTN registry (ISRCTN14024829), with registration finalised 8 weeks after the first randomised participant. No outcome data were available, no patients had completed follow-up and there was no change in data collection by the time of registration.

### Objective

The objective of the trial was to determine whether addition of PET/CT prior to CT-guided pleural biopsy increases its diagnostic sensitivity in patients with a previous non-diagnostic biopsy.

### Participants

Patients with a suspected pleural malignancy after a non-diagnostic biopsy were screened and recruited through the local lung cancer/mesothelioma multidisciplinary team (MDT) meetings where patients with suspected cancer undergoing investigation were discussed.

Patients were eligible if they met all of the following criteria: pleural thickening on CT suspicious for pleural malignancy; had any form of pleural biopsy (image guided or thoracoscopic) in the previous 12 months which was non-diagnostic; and if the lung cancer/mesothelioma MDT concluded there was sufficient concern for pleural malignancy to warrant a further pleural biopsy.

Patients were excluded if they met any of the following criteria: pleural thickening not amenable to a CT-guided biopsy; received talc pleurodesis within the previous 6 months; unsuitable for a CT-guided biopsy (*e.g.* unable to lie flat for the duration of the biopsy/uncorrectable coagulopathy); unable to give informed consent; pregnant or lactating; or aged <18 years.

### Interventions

Participants randomised to the intervention group had a PET-CT scan followed by a CT-guided biopsy. Participants in the comparator standard care group had a CT-guided biopsy only.

### Baseline assessment and follow-up

Patients who were eligible and consented had a baseline assessment prior to randomisation where demographic data, asbestos exposure history and previous biopsy information were recorded. A research-specific blood test for soluble mesothelin-related peptide (here on referred to as “mesothelin”) was performed alongside routine blood investigations. Follow-up was scheduled at 2 weeks post-biopsy, and 3, 6 and 12 months from randomisation. Patients recruited in the last 6 months of the trial were followed up to 6 months only.

### Outcomes

The primary outcome of the study was the proportion of correct diagnoses identified on the trial biopsy. A malignant diagnosis made from the trial biopsy was considered to be a correct diagnosis, as was a non-malignant diagnosis that was consistent to the end of the 12-month follow-up. If, during follow-up, a malignant diagnosis was made from sources other than the trial biopsy, then the latter was considered to be a false negative (see supplementary material for further details).

Secondary outcomes were defined in the protocol as: 1) total number of invasive procedures (video-assisted thoracic surgery (VATS)- or radiology-guided biopsies) undertaken following randomisation to confirm the diagnosis; 2) time from randomisation to cancer diagnosis (those not diagnosed with cancer were censored at last follow-up); 3) time from randomisation to death (survivors censored at last follow-up); 4) total number of hospital attendances following randomisation to confirm the diagnosis; 5) procedure-related adverse events; 6) uptake of chemotherapy following a positive diagnosis, during follow-up; 7) diagnostic utility of serum mesothelin levels measured at baseline, 6- and 12-month follow-up visits; and 8) PET scan parameters, in the PET-CT group (tumour glycolytic volume and standardised uptake values (SUVs)).

In addition we performed an exploratory analysis of how the PET-CT influenced the radiologist's decision making by changing the pleural biopsy site. The PET scan was also considered to have had impact if it indicated either an extrapleural biopsy site or an upstaging of disease. See supplementary material for further details.

### Sample size

In order to achieve 80% power (at 5% statistical significance) to detect a difference in the proportion of participants with a correct positive cancer diagnosis of 30% (from 20% in the CT-only group to 50% in the PET-CT group), the sample size was set at 78 (39 in each group). This effect size was consistent with pilot data from our institution. This sample size also provided 80% power to detect a doubling of the “hazard” (*i.e.* a hazard ratio (HR) of 2) for time to diagnosis or survival, assuming 5% dropout.

### Randomisation

Randomisation was performed using a web-based randomisation system developed by Bristol Trials Centre (Bristol, UK). Randomised allocations (1:1) were blocked, with varying block sizes, and stratified by centre. Allocations were concealed and not disclosed until a participant had been fully recruited.

### Blinding

Due to the nature of the trial procedures neither participants nor investigators were blinded to treatment allocation. Histopathologists interpreting the biopsy specimens were blinded to allocation and clinical details.

### Statistical methods

Analyses were based on a pre-specified statistical analysis plan (SAP) and performed on an intention-to-treat (ITT), or modified ITT, basis unless otherwise stated. Time-to-event outcomes were compared using Cox proportional hazards models (with death treated as a competing event), binary outcomes using generalised linear models and categorical (count) outcomes using Poisson regression (or negative binomial regression in cases of overdispersion). Model fit was assessed *via* standard methods (*e.g.* graphical plots) and if inadequate then alternative analysis methods were sought. All analyses used the CT-only group as the reference group and were adjusted for centre as a random effect (unless otherwise indicated). Outcomes are reported as effect sizes with 95% confidence intervals and likelihood ratio tests were used to determine statistical significance. Some outcomes (pre-specified in the SAP) were described but not formally compared (see supplementary material for details). Pre-specified sensitivity analyses included analyses excluding participants that did not receive the intervention allocated and imputing missing primary outcome data (see supplementary material for details). The ability of the serum mesothelin levels to predict a positive diagnosis (sensitivity, specificity, positive predictive value (PPV), negative predictive value (NPV) and area under the receiver operating curve (AUC)) were assessed for the study cohort as a whole. Analyses of the value of the PET scan parameters to predict a positive diagnosis were restricted to participants in the PET-CT group.

All analyses were performed in Stata version 15.1 (StataCorp, College Station, TX, USA). See supplementary material for further details.

## Results

**FIGURE 1:**
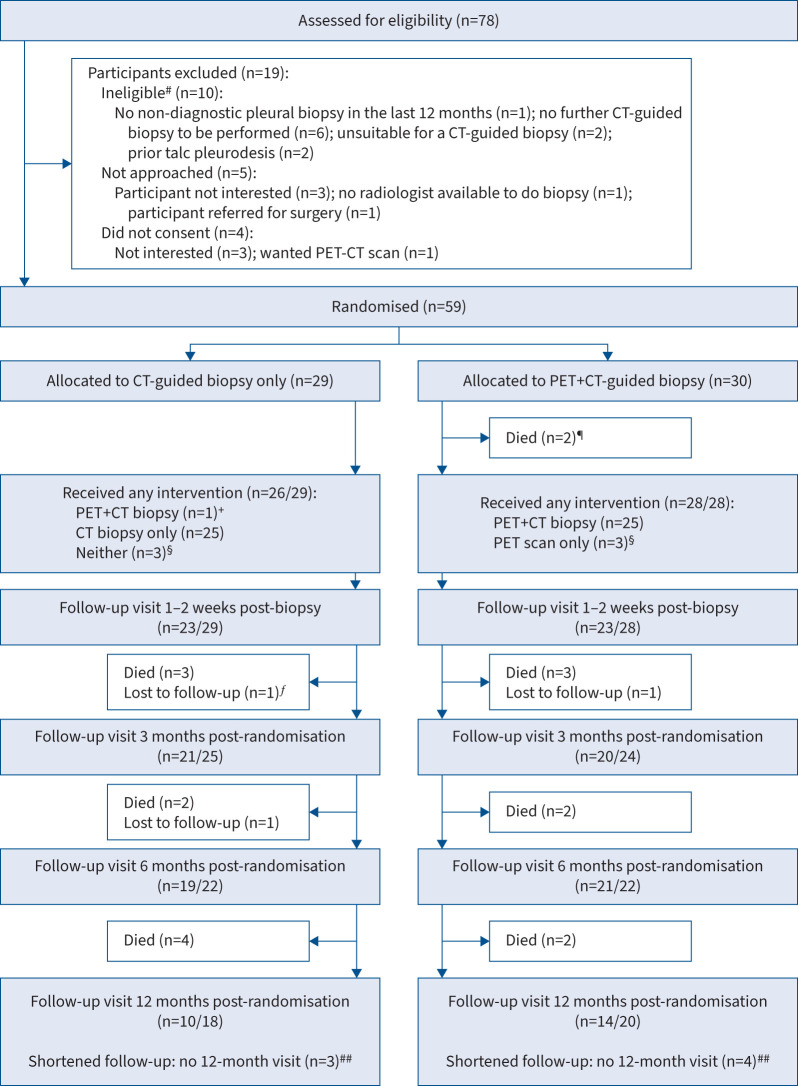
Flow of participants. Where follow-up visits were not attended (*e.g.* due to poor health), it was sometimes possible to obtain secondary outcome data from other sources (*e.g.* phone calls or patient notes). Primary outcome (diagnosis) data were not obtained from follow-up visits. ^#^: some patients may be ineligible for more than one reason; ^¶^: two participants were too unwell to attend their positron emission tomography-computed tomography (PET-CT) scan and CT biopsy appointments and died within 2 weeks of randomisation; ^+^: one participant randomised to CT biopsy only (“CT-only”) received a PET scan in addition due to a paraspinal lesion; ^§^: three participants failed to attend their biopsy appointment (one randomised to CT-only, two randomised to PET-CT and CT biopsy: “PET-CT”) and a biopsy was deemed inappropriate for three participants (two CT-only, one PET-CT); ^ƒ^: this participant had not received a malignant diagnosis prior to loss to follow-up; ^##^: 13 participants (six CT-only, seven PET-CT) in total were randomised during the extended recruitment period and could not have a 12-month follow-up visit: one (CT-only) was lost to follow-up and five (two CT-only, three PET-CT) died before 6 months.

Between September 2015 and September 2018, 78 patients from eight hospital sites (supplementary table S1) were screened for inclusion in the trial, 68 (87%) of whom were eligible (figure 1). 63 (93%) eligible patients were approached, and 59 (94%) consented and were randomised. 29 patients were randomised to the CT-only group (standard care) and 30 to the PET-CT group (intervention). 13 participants were recruited during the final year of the study and were followed up for just 6 months.

Participant demography and history are shown in table 1 and supplementary table S2. The median age was 75 years, and the majority of participants were male (51/59 (86%)) and had some history of asbestos exposure (50/59 (85%)). Overall, clinical characteristics were balanced between groups. In terms of previous interventions, the majority had had just one attempted biopsy prior to randomisation (51/59 (86%)). For 41% (12/29) of the CT-only group the previous pleural intervention had been percutaneous (*i.e.* CT/ultrasound-guided or closed biopsy) compared with 50% (15/30) of the PET-CT group; the remainder being thoracoscopic (*i.e.* local anaesthetic thoracoscopy or VATS).

**TABLE 1:** Baseline demographics and initial biopsy details

	**Randomised to CT-guided biopsy only (n=29)**	**Randomised to PET-CT and CT-guided biopsy (n=30)**	**Overall (n=59)**
**Age at randomisation (years)**	74 (69–80)	76 (71–82)	75 (70–80)
**Male**	26/29 (89.7)	25/30 (83.3)	51/59 (86.4)
**Known asbestos exposure**	24/29 (83)	26/30 (87)	50/59 (85)
**History of non-pleural respiratory disease**	10/29 (34)	6/30 (20)	16/59 (27)
**History of pleural disease**	10/29 (34)	8/30 (27)	18/59 (31)
**Most recent previous biopsy**			
CT guided	9/29 (31)	11/30 (37)	20/59 (34)
Ultrasound guided	3/29 (10)	3/30 (10)	6/59 (10)
LAT	13/29 (45)	9/30 (30)	22/59 (37)
VATS	4/29 (14)	6/30 (20)	10/59 (17)
Closed pleural	0/29 (0)	1/30 (3)	1/59 (2)
**Time since last biopsy (days)**	33 (24–63)	26 (14–41)	29 (19–47)
**Total number of previous biopsies**			
1	27/29 (93)	24/30 (80)	51/59 (86)
2	1/29 (3)	6/30 (20)	7/59 (12)
4	1/29 (3)	0/30 (0)	1/59 (2)

Data are presented as median (interquartile range) or n/N (%); where denominators do not match total expected numbers, this indicates missing data. CT: computed tomography; PET: positron emission tomography; LAT: local anaesthetic thoracoscopy; VATS: video-assisted thoracoscopic surgery.

### Adherence with study protocol

In total there were nine protocol deviations: four in the CT-only group and five in the PET-CT group. In the CT-only group, three participants did not have their CT-guided biopsy and one participant had a PET-CT prior to the CT-guided biopsy (a crossover). It was documented that the site of biopsy did not change on the basis of this PET-CT and the biopsy itself was inconclusive. In the PET-CT group, five participants did not have their CT-guided biopsy, two of whom also did not have the PET-CT scan (full details in figure 1, and supplementary tables S3 and S4). Biopsy details are summarised in supplementary table S5.

18 participants died during follow-up (nine in each group) and three participants (two CT-only, one PET-CT) were lost to follow-up between 2 weeks and 6 months of follow-up (figure 1). Of the 13 participants (six CT-only, eight PET-CT) followed up for 6 months only, seven (three CT-only, four PET-CT) were still in the trial at 6 months and five (two CT-only, three PET-CT) had not received a malignant diagnosis by this time.

### Primary outcome

In total, the study biopsy yielded 43 correct diagnoses (including confirmed benign diagnoses): 22/26 (85%) in the CT-only group and 21/24 (88%) in the PET-CT group; risk ratio 1.03 (95% CI 0.83–1.29) and risk difference 0.03 (95% CI −0.16–0.22) (p=0.77) (table 2). Overall, 28 diagnoses of pleural malignancy were made from the study biopsy: 15 in the CT-only group and 13 in the PET-CT group (intervention). Excluding participants who did not receive the allocated intervention provided the same conclusion (supplementary table S6).

**TABLE 2:** Primary outcome: diagnostic sensitivity of trial biopsy

	**Randomised to CT-guided biopsy only (n=29)**	**Randomised to PET-CT and CT-guided biopsy (n=30)**	**Effect size (95% CI)**	**p-value**
**Primary outcome**				
Diagnostic sensitivity of trial biopsy	22/26 (84.6)	21/24 (87.5)	RR 1.03 (0.83–1.29) RD 0.03 (−0.16–0.22)	0.77 0.77
Pleural malignancy diagnosed from trial biopsy	15/29 (51.7)	13/30 (43.3)		
**Final diagnoses**				
Pleural malignancy diagnosed by 6-month follow-up	19/28 (67.9)	14/28 (50.0)		
Pleural malignancy diagnosed by 12-month follow-up	19/26 (73.1)	16/24 (66.7)		
Overall final diagnosis				
Epithelioid mesothelioma	12/29 (41.4)	9/30 (30.0)		
Sarcomatoid mesothelioma	3/29 (10.3)	2/30 (6.7)		
Biphasic mesothelioma	2/29 (6.9)	1/30 (3.3)		
Mesotheliomas not otherwise specified	1/29 (3.4)	1/30 (3.3)		
Desmoplastic mesothelioma	1/29 (3.4)	0/30 (0.0)		
Lung adenocarcinoma	0/29 (0.0)	2/30 (6.7)		
Follicular lymphoma	0/29 (0.0)	1/30 (3.3)		
Benign/no malignant diagnosis/no trial biopsy	10/29 (34.5)	14/30 (46.7)		

Data are presented as n (%), unless otherwise stated; where denominators do not match total expected numbers, this indicates missing data. CT: computed tomography; PET: positron emission tomography; RR: risk ratio; RD: risk difference. For the primary outcome, three CT arm and six PET-CT arm patients are missing diagnostic sensitivity of the trial biopsy as they did not receive a malignant diagnosis from the trial biopsy and we were not able to assign them a true negative diagnosis as they were not followed up for the full 12 months, due to death, loss to follow-up or shortened follow-up. The latter missing data were considered to be missing at random and imputed to produce the effect size estimate shown.

The sensitivity of the trial biopsy to correctly identify pleural malignancy was 79% (95% CI 54–94%) in the CT-only group with a NPV of 64% (95% CI 31–89%). This compared with a sensitivity of 81% (95% CI 54–96%) and a NPV of 73% (95% CI 39–94%) in the PET-CT group (supplementary table S7).

In the CT-only group, a diagnosis of pleural malignancy varied depending on the nature of the previous biopsy mode. If a percutaneous biopsy (*i.e.* CT- or ultrasound-guided biopsy) had been performed previously then the chance of correct identification of pleural malignancy was 66% (8/12); if the biopsy was thoracoscopic then the likelihood was 82% (14/17). In the PET-CT group, if a percutaneous biopsy had been performed previously then the chance of a correct identification of pleural malignancy was 80% (12/15) compared with 82% (9/15) if the biopsy was thoracoscopic.

### Secondary outcomes: time from randomisation to cancer diagnosis and death

The median time to pleural malignancy diagnosis was longer for participants in the PET-CT group (92 days) compared with the CT-only group (35 days) (sub-HR (SHR) treating death as a competing event 0.65 (95% CI 0.32–1.33); p=0.24) (table 3). An ancillary analysis of the time to a correct diagnosis (from the study biopsy or any subsequent biopsy within the 12-month follow-up period) gave a similar result (SHR 0.72 (95% CI 0.40–1.30); p=0.28). Survival was similar in the two groups: median 221 *versus* 255 days (HR 1.04 (95% CI 0.39–2.75); p=0.94) in the CT-only and PET-CT groups, respectively.

**TABLE 3:** Secondary outcomes: mortality, invasive procedures, hospital attendances, chemotherapy uptake and adverse events

	**Randomised to CT-guided biopsy only (n=29)**	**Randomised to PET-CT and CT-guided biopsy (n=30)**	**Effect^#^** ** (95% CI)**	**p-value**
**Time to diagnosis of pleural malignancy (days)**	35 (20.0–168.0)	92 (21.0–375.0)	SHR 0.65 (0.32–1.33)	0.24
**Patient died**	9/29 (31)	9/30 (30)		
**Time to death (days)**	221 (183–360)	255 (150–373)	HR 1.04 (0.39–2.75)	0.94
**Total invasive procedures undertaken to confirm diagnosis** **^#^**	1.0 (1.0–1.0)	1.0 (1.0–1.0)	IRR 0.93 (0.56–1.55)	0.78
**Total hospital attendances undertaken to confirm diagnosis** **^¶^**	0.0 (0.0–1.0)	0.5 (0.0–2.0)	IRR 1.30 (0.54–3.13)	0.56
**Uptake of chemotherapy**	5/17 (29)	5/15 (33)		
**Time to uptake of chemotherapy (days)** **^+^**	188.0 (73.0–267.0)	114.0 (48.0–308.0)		
**Total number of procedure-related adverse events**				
0	20/24 (83.3)	21/23 (91.3)		
1	4/24 (16.7)	1/23 (4.3)		
2	0/24 (0.0)	1/23 (4.3)		

Data are presented as median (interquartile range) or n/N (%), unless otherwise stated; where denominators do not match total expected numbers, this indicates missing data. CT: computed tomography; PET: positron emission tomography; SHR: sub-hazard ratio; HR: hazard ratio; IRR: incident rate ratio. ^#^: data missing for one patient (n=1 CT-only); ^¶^: data missing for four patients (n=2 CT-only, n=2 PET-CT); ^+^: data missing for three patients (n=2 CT-only, n=1 PET-CT).

### Secondary outcomes: further invasive diagnostic procedures, anti-cancer treatment uptake and hospital attendances

The number of invasive procedures (including the trial biopsy) undertaken to confirm the diagnosis was similar in each group, with most participants having a single procedure (incidence rate ratio (IRR) 0.93 (95% CI 0.56–1.55); p=0.78) (table 3 and supplementary table S8). The number of hospital visits, following trial biopsy, to confirm the diagnosis was also similar (median 0 *versus* 0.5 in the CT-only and PET-CT groups: IRR 1.30 (95% CI 0.54–3.13); p=0.56). At follow-up, 10 patients (10/32 (31%)) with confirmed malignancy had taken up some form of chemotherapy (table 3). Median time (days) to uptake of treatment was lower in those randomised to the PET-CT group compared with CT-only (114 *versus* 188 days) (table 3). Further details of the chemotherapy and radiotherapy received are given in supplementary table S10.

### Secondary outcomes: PET scan parameters and additional findings of PET-CT

The maximal SUV of the pleura was captured for 24 patients randomised to PET-CT. The median maximum SUV at baseline was 6.3 (supplementary table S8). Categorising maximum SUV at baseline into low (≤7.4) or high (>7.4), with the cut-point chosen to maximise the AUC, gave a sensitivity of 77% (95% CI 46–95%) and specificity of 91% (95% CI 59–100%) to predict diagnosis of pleural malignancy compared with using a clinically defined cut-point of 3.5, which gave a sensitivity of 92% (95% CI 64–100%) and specificity of 36% (95% CI 11–69%) (supplementary table S9c and d) [7].

Of the patients with a correct diagnosis from the trial biopsy, the PET scan impacted the diagnostic pathway in 14/21 patients: it led to upstaging in 2/21 patients (9.5%), a biopsy site change in 8/21 patients (38.1%), and both upstaging and biopsy site change in 4/21 patients (19.0%) (supplementary table S11).

An analysis of the PET reports (n=27) identified seven (26%) incidental findings, *i.e.* definite or suspected pathology unrelated to the primary reason for the scan, none of which led to any change in the patients’ management: thyroid goitre (n=3), renal calculus (n=1), adrenal nodule (n=2) and rectal polyp (n=1).

### Secondary outcome: mesothelin

Using the cut-off at 2.0 nmol·L^−1^ serum mesothelin had a sensitivity of 73%, specificity of 78%, PPV of 82%, NPV of 69% and AUC of 0.83 for predicting the diagnosis of pleural mesothelioma in the entire cohort (see supplementary tables S9a and b and S12 for full details).

### Adverse events

There were seven adverse events related to the study procedures, all relating to the biopsy itself: four in the CT-only group and three in the PET-CT group (supplementary table S13). Pain at the biopsy site was experienced by five participants (three CT-only, two PET-CT), bleeding and bruising by one patient (PET-CT) and pneumothorax by one patient (CT-only). No expected events related to the PET scan were observed.

During the 12-month follow-up period a total of 82 adverse events (46 CT-only, 36 PET-CT) were recorded in 36 participants (19 CT-only, 17 PET-CT) (see supplementary table S13 for further details). Of these, 56 events (32 CT-only, 24 PET-CT) in 29 patients (15 CT-only, 14 PET-CT) were classed as serious (*i.e.* complications that were life threatening or caused hospitalisation, increased length of hospital admission, persistent or significant disability, or death).

## Discussion

This is the first RCT to investigate the added benefit of PET-CT for patients requiring a repeat biopsy for pleural thickening suspicious for malignancy. For the primary outcome of diagnostic sensitivity of the subsequent biopsy, the addition of PET-CT (intervention) had similar rates to CT-guided biopsy alone (standard care). There was no evidence to suggest PET-CT was superior to CT-guided biopsy alone; the rate was just 3% higher in the PET-CT group. However, the sample size was small and the 95% confidence interval suggests the difference could be between 16% lower and 22% higher with PET-CT. Despite this uncertainty, this interval excludes the target 30% difference the trial was designed to detect. Moreover, had the trial recruited to target and similar rates of correct diagnosis from the second biopsy were seen in these additional participants, an ancillary analysis repeating the primary outcome analysis imputing data for these extra participants gave a relative risk of 1.09 (95% CI 0.89–1.32) and a risk difference of 0.07 (95% CI −0.09–0.23) (p=0.39), consistent with our primary analysis.

The utility of PET-CT in the diagnostic pathway of other malignancies is well established, playing a central role in lung cancer diagnosis and management [8]. It does, however, have distinct disadvantages, including difficulty differentiating between malignancy and other metabolically active conditions such as infection or autoimmune disease. For this reason, patients who have had previous talc pleurodesis may have abnormal pleural uptake for many years post-procedure [9]. In addition, PET-CT has a well-established rate of incidental findings which could represent management altering early-stage malignancies that can be difficult to manage/investigate especially in the context of possible pleural malignancy. Finally, the availability of PET scanners varies dramatically worldwide so any guidelines should take this into account [10].

The role of PET-CT in pleural disease is much less defined than other solid organ malignancies. The focus of previous studies has been on its role in initial diagnosis and prognostication. Porcel *et al.* [11] performed a meta-analysis of diagnostic accuracy studies focusing specifically on PET for malignant pleural disease. From 14 studies (comprising 407 patients with malignant disease) the pooled test characteristics of PET imaging had a sensitivity of 81% and specificity of 74%. The studies included were highly heterogenous and the authors concluded that there was no basis for the routine inclusion of PET in the diagnosis of malignant pleural disease, which concurs with international guidelines [12].

On the role of PET in prognosticating pleural mesothelioma, where CT imaging is less helpful, the evidence is not conclusive [7, 13]. Depending on the PET end-point used some studies have shown a role in baseline prognostication but no ability to monitor disease [7]. The 2018 British Thoracic Society guidelines on pleural mesothelioma do not recommend the routine use of PET-CT for suspected pleural malignancy except in patients “where excluding distant metastases will change management” [14].

The TARGET trial assessed a novel use of PET-CT in the diagnosis of pleural malignancy to potentially guide radiologists to sites of high uptake. This approach is theoretically viable as pleural malignancy, especially mesothelioma, is very heterogenous, which cannot be appreciated using CT alone [15]. Furthermore, the proportion of participants with a correct positive diagnosis on the trial biopsy was notably higher in the CT-only group (51%) than the 20% assumed in the power calculation and by chance was higher than the 43% observed in the PET-CT group. It suggests that if there remains a high clinical suspicion of malignancy with pleural thickening after a single inconclusive pleural biopsy it is worthwhile repeating a CT-guided biopsy as the chance of successful diagnosis is high.

This trial did demonstrate that some additional useful information can be generated from a PET-CT. In multifocal disease a biopsy can be targeted to an area that is easily accessible, such as an axillary node biopsy, rather than pleural biopsy that can be more invasive. However, this should be balanced against a significant rate of incidental findings detected in the PET-CT group, with over a quarter of participants having findings that required further imaging or MDT discussion. In addition, while median survival from randomisation was similar between the two groups (221 *versus* 255 days), the median time to pleural malignancy diagnosis was longer for participants in the PET-CT group compared with the CT-only group (92 *versus* 35 days), suggesting addition of a PET-CT leads to delays to the diagnostic pathway.

This trial also assessed the role of mesothelin, a membrane-bound glycoprotein overexpressed by malignant mesothelial cells [16]. Soluble mesothelin is found in the blood and pleural fluid of patients with pleural mesothelioma and levels correlate with tumour stage and bulk. It lacks the sensitivity to be used as a diagnostic marker given reduced expression in non-epithelioid pleural mesothelioma. Using a cut-off of 2.0 nmol·L^−1^, the sensitivity of mesothelin to predict pleural mesothelioma was 73% with a specificity of 78%. A meta-analysis of 28 studies by Cui *et al*. [17] demonstrated that serum mesothelin had a pooled sensitivity and specificity of 61% and 87%, respectively, to predict pleural mesothelioma, similar to results from this trial (sensitivity of 73% and specificity of 78% to predict pleural mesothelioma), too low to have utility as a stand-alone diagnostic test (supplementary table S9a).

This trial has strengths and weaknesses. Strengths include the randomised design allowing an unbiased comparison between PET-CT and CT-only. This strength remains despite the trial failing to reach its recruitment target. Another strength was the blinding of histopathologists interpreting the biopsy specimens. Limitations include the recruitment being lower than the target, reducing the power to detect a difference between groups, and having less than 12 months follow-up on participants recruited towards the end of the recruitment period. The sample size was not met due to changes in the national diagnostic pathway (such as increasing provision of VATS biopsy) and changes in histopathological techniques. There have been several advances in the field of pleural mesothelioma diagnosis, with BAP1 loss and p16 fluorescence *in situ* hybridisation increasing the sensitivity and specificity of biopsies and pleural fluid [18]. As a result, the proportion of patients requiring a repeat biopsy has changed from when this trial was recruiting. There was considerable patient attrition during follow-up due to death, which reflects the aggressive nature of pleural mesothelioma and cancers that have metastasised to the pleura. It affirms that trying to shorten the diagnostic pathway in these individuals is important given their limited life expectancy.

### Conclusions

This trial, although under-recruited, suggests there is no benefit in conducting a routine PET/CT scan in patients with initial inconclusive pleural biopsies, and potential harms in this approach include longer diagnostic pathway and incidental findings. CT-guided biopsy alone has a sufficiently high diagnostic yield in this cohort of patients to remain the optimal diagnostic method.

## Supplementary material

10.1183/13993003.01295-2023.Supp1 **Please note:** supplementary material is not edited by the Editorial Office, and is uploaded as it has been supplied by the author.
Supplementary material ERJ-01295-2023.Supplement

## Shareable PDF

10.1183/13993003.01295-2023.Shareable1 This one-page PDF can be shared freely online.
Shareable PDF ERJ-01295-2023.Shareable
