# Supplementary material for: PET-CT-guided versus CT-guided biopsy in suspected malignant pleural thickening: a randomised trial
Source: Eur Respir J. 2024 Feb 1;63(2):2301295. doi: 10.1183/13993003.01295-2023 (PMC10831139; doi:10.1183/13993003.01295-2023)
Supplement: Supplementary file 1 [file ERJ-01295-2023.Supplement.pdf]

SUPPLEMENTARY MATERIALS

Supplementary methods

Primary outcome definition: further detail

To determine the primary outcome of correct diagnosis of pleural malignancy on the second biopsy, participants were followed-up for 12 months (or to the end of the trial for participants recruited in the last 6 months). If a diagnosis of pleural malignancy was made from the second biopsy, this was classified as a correct diagnosis from the second biopsy. Participants not diagnosed with pleural malignancy on second biopsy (i.e. with a second non-diagnostic biopsy) had interval imaging at 3, 6 and 12 months (where possible) and possibly a further biopsy considered. A diagnosis of pleural malignancy during follow-up may be based on a further biopsy(s) or clinic-radiological findings (e.g. due to characteristic progressive features on imaging or deteriorating modified RECIST score). A diagnosis of pleural malignancy (from sources other than the second biopsy) made at any MDT meeting during follow-up was considered as a definitive diagnosis and the second biopsy as giving an incorrect diagnosis. If no diagnosis of pleural malignancy was made by the end of 12 months follow-up the final diagnosis was classified as non-malignant and the second biopsy as giving a correct diagnosis.

The definition of the primary outcome is summarised below:-

**Outcome of second biopsy**	**Outcome at 12 months**	**Correct diagnosis on second biopsy**
Pleural malignancy confirmed	Pleural malignancy confirmed	Yes, true positive
Biopsy non-diagnostic	Non-malignant confirmed	Yes, true negative
	Pleural malignancy confirmed	No, false negative

Supplementary statistical methods

Formal comparison of outcomes

The Statistical Analysis Plan pre-specified that formal comparison between treatment groups would not be carried out for certain outcomes, in order to minimise type I errors, these were a). time to uptake of chemotherapy data which was only applicable to a subset of randomised patients, hence the balance between groups achieved by randomisation would no longer exist (and the size of the patient subset was likely to be too small for meaningful comparison) and b) the number of procedure-related adverse events which was expected to be low and hence not appropriate for formal comparison.

Shortened follow-up

Due to slower recruitment than expected as a result of changing clinical practice during the course of the study, the planned recruitment period was extended by 12 months but with a reduced follow-up of 6 months for patients recruited in the last 6 months. In order to take this into account in the statistical analysis, binary or count outcomes were analysed using a time to event or rate comparison approach where possible. This was not possible for the primary outcome, as data at 12 months were needed to determine whether the absence of a malignant diagnosis from the study biopsy represented a true benign diagnosis: these missing data (due to shortened follow-up by study design) were handled using multiple imputation methods (see below).

Missing data

As it was not possible to follow-up all participants recruited in the last 6 months of the trial for the full 12 months, participants without a malignant diagnosis recorded by the end of follow-up were missing their primary outcome data (as a non-malignant diagnosis from the study biopsy could not be confirmed). Multiple imputation using chained equations (with 10 imputed datasets) was used to

handle these missing data. The imputation model contained treatment allocation group, baseline mesothelin level and diagnosis data up to 6 months follow-up: it was not possible to include site in the imputation model due low numbers in several sites and hence primary outcome analysis models using imputed datasets did not adjust for site. To determine the impact of missing primary outcome data not by design in 3 patients (1 CT-only and 2 PET-CT) due to death before the second biopsy or loss to follow-up at an early stage, sensitivity analyses were carried out, a) Infilling with 'incorrect diagnosis' and b) infilling with 'correct diagnosis'.

A complete case analysis approach was used for other outcomes, as levels of missing data were less than 7% for all outcomes that were formally compared by allocation group,

Binary outcomes

Generalised linear models (binomial and log-binomial) were used to estimate relative risk and risk difference for the primary outcome (correct diagnosis from the second biopsy). Iteratively reweighted least squares was used in analysis models estimating relative risk due to convergence issues.

Time to event outcomes

Time from randomisation to diagnosis of pleural malignancy and from randomisation to death were analysed using Cox's proportional hazard models stratified by site. Patients not experiencing the outcome in question (diagnosis or death) were censored at the time of their last follow-up. For time to diagnosis, death was modelled as a competing risk in a model stratified by site by modelling cause-specific cumulative incidence functions using time-dependent weights and comparison of the corresponding subdistribution hazards (or sub-hazards) according to Fine and Grey's propotional sub-hazards model [1].

Continuous outcomes

Continuous data were summarised using mean and standard deviation (or median and interquartile range (IQR) if distributions were skewed. The ability of baseline serum mesothelin levels to predict a diagnosis of pleural malignancy was assessed for the study cohort as a whole. Similar analyses of the value of the PET scan maximum SUV to predict a diagnosis of pleural malignancy was restricted to the PET-CT group. Logistic regression models used to calculate the area under the Receiver Operating Characteristics (ROC) curve were adjusted for age, sex, presence of respiratory disease and presence of pleural disease as fixed effects, in addition to adjusting for site as a random effect. It was not possible to adjust for duration of asbestos exposure due to high levels of missing data. To determine sensitivity, specificity, positive and negative predictive values a clinically chosen cut-off was used: 2.0 for mesothelin and 3.5 for maximum SUV (latter added as an anciliary/post-hoc analysis). Due to variation in the literature concerning the optimal cut-off the analysis of baseline maximum SUV was also performed using the point nearest to (0,1) on the ROC curve (giving a cut-off value of 7.4). In the case of prediction of pleural malignancy by baseline mesothelin, one outlier led to poor model fit and was hence omitted from the logistic regression model used to calculate the area under the curve.

Count outcomes

Count data were summarised using a number and percentage. Two count outcomes were formally compared by allocation group: the number of invasive procedures undertaken to confirm the diagnosis and the number of hospital attendances to confirm the diagnosis. Invasive procedures or hospital attendances were considered to have contributed to confirming the diagnosis if they were recorded at or before the follow-up timepoint when the diagnosis was made. The analysis approach that was pre-specified in the SAP for these count outcomes was Poisson regression, using the duration of follow-up as the exposure variable, as participant attrition during follow-up was anticipated (due to deaths associated with the poor prognosis of mesothelioma, loss to follow-up

and a reduction in the maximum follow-up period from 6 to 12 months for 14 patients in order to extend the recruitment period). However, the distribution of the number of outpatient visits made prior to diagnosis showed overdispersion and so was modelled using negative binomial regression rather than Poisson regression. Analyses were adjusted for site as a random effect.

Impact on diagnostic pathway

While the study was in progress a new binary outcome was added to the Statistical Analysis Plan: how the PET scan impacted clinically. Independent radiological review of the PET scan imaging was undertaken in order to determine whether the PET 'ead to upstaging of the disease and whether the PET scan suggested a biopsy site change (including a site change to 'none' i.e. no biopsy).

Sensitivity analyses

To assess the effect of patients not receiving the allocated intervention, all analyses were repeated omitting patients that did not have their complete allocated intervention (CT-biopsy only or PET-CT scan and CT-biopsy). In addition, a further sensitivity analysis were pre-specified for the primary outcome, namely using a best/worst case simple imputation approach for patients missing primary outcome data due to death, withdrawal or loss to follow-up before the intervention took place.

Ancillary (post-hoc) analyses

An ancillary analysis was carried out to determine the specificity and sensitivity of baseline SUV in prediction of pleural malignancy diagnosis using a clinically-defined cut point of 3.5 (rather than a data-defined cut point: see *continuous outcomes* section).

Supplementary Tables

Table S1: Screening data by centre

	Screened	EXCLUDED FROM STUDY				Randomised	
		Ineligible	Not approached	Did not consent	Other reason	(n)	(% of screened patients)
Bristol	31	2	0	1	0	28	90
Glasgow	10	2	2	0	0	6	60
Gloucester	4	0	0	1	0	3	75
Newport	6	1	0	0	0	5	83
Norfolk & Norwich	4	2	1	0	0	1	25
Oxford	11	0	1	1	0	9	82
Sheffield	9	3	1	1	0	4	44
Stoke	3	0	0	0	0	3	100
**TOTAL**	78	10	5	4	0	59	75

Table S2: Patient demography and past history (all randomised patients): further detail

		Randomised to CT guided biopsy only (n=29)		Randomised to PET-CT and CT guided biopsy (n=30)		Overall (n=59)	
		n	%	n	%	n	%
DEMOGRAPHY							
Weight (kg) [1]	(median, IQR)	71	(63, 85)	70	(63, 80)	70	(63, 83)
ASBESTOS EXPOSURE HISTORY							
Known asbestos exposure		24/29	83%	26/30	87%	50/59	85%
Dock worker		0/24	0%	3/26	12%	3/50	6%
Plumber		0/24	0%	0/26	0%	0/50	0%
Electrician		1/24	4%	1/26	4%	2/50	4%
Teacher		1/24	4%	1/26	4%	2/50	4%
Builder		4/24	17%	4/26	15%	8/50	16%
Rail works		1/24	4%	0/26	0%	1/50	2%
Joiner		3/24	13%	1/26	4%	4/50	8%
Para-exposure		2/24	8%	0/26	0%	2/50	4%
Other		15/24	63%	17/26	65%	32/50	64%
Duration of symptoms (years) [2]	(median, IQR)	6	(3, 12)	5	(1, 9)	5	(3, 9)
RESPIRATORY DISEASE (NON-PLEURAL)							
Non-pleural disease OVERALL		10/29	34%	6/30	20%	16/59	27%
Asthma		4/10	40%	3/6	50%	7/16	44%
Chronic obstructive pulmonary disease		5/10	50%	3/6	50%	8/16	50%
Interstitial lung disease		1/10	10%	0/6	0%	1/16	6%
Previous tuberculosis		0/10	0%	0/6	0%	0/16	0%
Other non-pleural disease		0/10	0%	0/6	0%	0/16	0%
PLEURAL DISEASE							
Pleural disease OVERALL		10/29	34%	8/30	27%	18/59	31%
Empyema		1/10	10%	2/8	25%	3/18	17%
Empyema laterality							
Left		0/1	0%	1/2	50%	1/3	33%
Right		1/1	100%	1/2	50%	2/3	67%
Time since diagnosis (years)	(median, IQR)	1.2	(1.2, 1.2)	0.6	(0.3, 1.0)	1.0	(0.3, 1.2)
Pneumothorax		5/10	50%	0/8	0%	5/18	28%
Pneumothorax laterality							
Left		2/5	40%			2/5	40%
Right		3/5	60%			3/5	60%
Time since diagnosis (years)	(median, IQR)	3.0	(1.2, 3.0)			3.0	(1.2, 3.0)
Chylothorax		0/10	0%	0/8	0%	0/18	0%
Benign asbestos-related pleural effusion		2/10	20%	2/8	25%	4/18	22%
Effusion laterality							
Left		1/2	50%	2/2	100%	3/4	75%
Right		1/2	50%	0/2	0%	1/4	25%
Time since diagnosis (years)	(median, IQR)	3.2	(2.0, 4.4)	3.8	(2.5, 5.1)	3.4	(2.2, 4.8)
Diffuse pleural thickening		6/10	60%	4/8	50%	10/18	56%
Thickening laterality							
Left		1/6	17%	3/4	75%	4/10	40%

		Randomised to CT guided biopsy only (n=29)		Randomised to PET-CT and CT guided biopsy (n=30)		Overall (n=59)	
		n	%	n	%	n	%
Right		4/6	67%	1/4	25%	5/10	50%
N/A		1/6	17%	0/4	0%	1/10	10%
Time since diagnosis (years)	(median, IQR)	1.5	(1.2, 2.8)	1.2	(0.9, 3.2)	1.3	(1.0, 2.8)
Previous thoracic surgery		4/10	40%	3/8	38%	7/18	39%
Surgery laterality							
Left		1/4	25%	2/3	67%	3/7	43%
Right		3/4	75%	1/3	33%	4/7	57%
Time since surgery (years)	(median, IQR)	1.5	(0.2, 4.1)	1.0	(0.3, 9.4)	1.0	(0.2, 5.4)
OTHER COMORBIDITY							
Coronary artery bypass graft		2/29	7%	0/30	0%	2/59	3%
Time since surgery (years)	(median, IQR)	11.6	(2.4, 20.8)			11.6	(2.4, 20.8)
Diabetes		1/29	3%	3/30	10%	4/59	7%
Diabetes controlled by	Diet	0/1	0.0%	1/3	33.3%	1/4	25.0%
	Tablets	1/1	100.0%	0/3	0.0%	1/4	25.0%
	Insulin	0/1	0.0%	2/3	66.7%	2/4	50.0%
On anticoagulation treatment		6/29	21%	11/30	37%	17/59	29%
Anticoagulant	Clopidrel	2/6	33.3%	3/11	27.3%	5/17	29.4%
	Warfarin	0/6	0.0%	1/11	9.1%	1/17	5.9%
	Rivaroxaban	2/6	33.3%	2/11	18.2%	4/17	23.5%
	Dabigatran	0/6	0.0%	1/11	9.1%	1/17	5.9%
	Apixaban	2/6	33.3%	4/11	36.4%	6/17	35.3%
BLOOD TEST RESULTS							
Haemoglobin (g/l)	(mean, SD)	127	17	132	15	129	16
Platelets (10^9/L)	(mean, SD)	366	124	368	127	367	124
Sodium (mmol/L) [3]	(mean, SD)	139	3	138	3	138	3
Potassium (mmol/L) [4]	(mean, SD)	4.5	0.4	4.5	0.5	4.5	0.4
Creatinine (umol/L) [5]	(mean, SD)	84	23	83	16	83	20
Estimated glomerular filtration rate (ml/min/1.73^2) [6]	(median, IQR)	77	(63, 87)	77	(66, 85)	77	(64, 86)
Urea (mmol/L) [7]	(mean, SD)	6.4	3.5	6.0	1.7	6.2	2.7
Prothrombin Time (s) [8]	(median, IQR)	11	(11, 13)	11	(11, 13)	11	(11, 13)
Activated partial thromboplastin time (s) [9]	(median, IQR)	28	(25, 31)	27	(25, 31)	28	(25, 31)
International normalised ratio [10]	(median, IQR)	1.0	(1.0, 1.1)	1.0	(1.0, 1.1)	1.0	(1.0, 1.1)
SPIROMETRY							
Spirometry performed		28/29	97%	25/30	83%	53/59	90%
If no, reason	Did not attend	0/1	0%	1/5	20%	1/6	17%
	Unable to perform	1/1	100%	1/5	20%	2/6	33%
	Performed but poor technique	0/1	0%	1/5	20%	1/6	17%
	Not appropriate today	0/1	0%	2/5	40%	2/6	33%

		Randomised to CT guided biopsy only (n=29)		Randomised to PET-CT and CT guided biopsy (n=30)		Overall (n=59)	
		n	%	n	%	n	%
Forced expiratory volume in 1 second (FEV1) (L) [11]	(mean, SD)	1.8	0.7	1.8	0.6	1.8	0.7
Forced vital capacity (FVC) (L) [12]	(mean, SD)	2.5	1.0	2.6	0.8	2.5	0.9
FEV1/FVC ratio (%)[13]	(mean, SD)	76	19	72	14	74	17
FEV1 % predicted [14]	(mean, SD)	69	21	77	19	73	21
FVC % predicted [15]	(mean, SD)	63	19	70	16	66	18

Note: Where denominators do not match total expected numbers, this indicates missing data. CT=computed tomography, PET=positron emission tomography, IQR=inter-quartile range, SD=standard deviation, FEV1= Forced expiratory volume in 1 second , FVC= Forced vital capacity.

[1] Data missing for 2 patients (1,1), [2] Data missing for 10 patients (6, 4), [3] Data missing for 1 patients (1, 0), [4] Data missing for 1 patients (1, 0), [5] Data missing for 2 patients (1, 1), [6] Data missing for 5 patients (3, 2): of these, 1 was recorded as >60 (CT-biopsy only group), 3 were recorded as >90 (2 CT-biopsy only group, 1 PET-CT group), [7]Data missing for 3 patients (3, 0), [8] Data missing for 8 patients (5, 3), [9] Data missing for 7 patients (5, 2), [10] Data missing for 7 patients (4, 3), [11] Data missing for 7 patients (2, 5), [12] Data missing for 7 patients (2, 5), [13] Data missing for 7 patients (2, 5), [14] Data missing for 8 patients (3, 5), [15] Data missing for 7 patients (2, 5).

Table S3: Protocol deviations (out of all randomised patients)

	Randomised to CT guided biopsy only (n=29)		Randomised to PET-CT and CT guided biopsy (n=30)		Overall (n=59)	
	Patients	%	Patients	%	Patients	%
**Any protocol deviation**[1]	4/29	13.8%	5/30	16.7%	9/59	15.3%
Patient randomised to CT-guided biopsy only, had PET-CT and CT-guided biopsy (crossover)	1/29	3.4%			1/29	3.4%
Patient randomised to CT-guided biopsy only, did not have CT-guided biopsy	3/29	10.3%			3/29	10.3%
Patient randomised to PET-CT and CT-guided biopsy, had PET-CT but not CT-guided biopsy			3/30	10.0%	3/30	10.0%
Patient randomised to PET-CT and CT-guided biopsy, had neither PET-CT nor CT-guided biopsy			2/30	6.7%	2/30	6.7%
Patient ineligible but treated in the study	0/29	0.0%	0/30	0.0%	0/59	0.0%

Note: CT=computed tomography, PET=positron emission tomography.

[1] In addition, 15 patients had more than 7 days from randomisation to PET-CT scan (range: 8 to 36 days)

1 patient had less than 2 days from PET-CT scan to CT-biopsy, though PET-CT scan results were available for their CT- biopsy

26 patients (10,16) had more than 14 days from randomisation to CT-biopsy (range: CT biopsy only: 15 to 67 days, PET and CT biopsy: 15 to 48 days)

1 patient did not have blood clotting screen prior to CT-guided biopsy

8 patients did not have a FEV1% predicted value available at baseline (3 randomised to CT biopsy only, 5 randomised to PET-CT and CT guided biopsy)

Table S4: Details of protocol deviations (out of all randomised patients)

Centre	Further details
Patient randomised to CT-guided biopsy only, had PET-CT and CT-guided biopsy (crossover)	
Stoke	The radiologist who agreed to do biopsy originally decided not to do because he felt the lesion was reducing in size. Because the lesion was paraspinal the radiologist was not confident and wanted to get a PET-CT. The site of biopsy did not change (documented in CRF). The biopsy was inconclusive. Clinically patient started to deteriorate and decided not to have a further biopsy
Patient randomised to CT-guided biopsy only, did not have CT-guided biopsy	
Newport	Did not attend CT-biopsy
Oxford	Subject attended the hospital for a CT-Guided biopsy. On assessment, it was decided by the radiologist in charge of the procedure that there was "no change to right sided pleural thickening, and thus now new site to biopsy." The procedure was thus not performed.
Sheffield	MDT error. Insufficient pleural thickening for CT guided biopsy.
Patient randomised to PET-CT and CT-guided biopsy, had PET-CT but not CT-guided biopsy	
Glasgow	Did not attend CT-biopsy.
Bristol	Radiologist felt that there had been an improvement in pleural thickening- did not feel biopsy appropriate
Oxford	Did not attend CT-biopsy
Patient randomised to PET-CT and CT-guided biopsy, had neither PET-CT nor CT-guided biopsy	
Newport	Too unwell. Patient died 11 days after randomisation.
Sheffield	Patient died 9 days after randomisation.

Note: CT=computed tomography, PET=positron emission tomography, MDT=multi-disciplinary team.

Table S5: Intervention details (all randomised patients)

		Randomised to CT guided biopsy only (n=29)		Randomised to PET-CT and CT guided biopsy (n=30)		Overall (n=59)	
		**n**	**%**	**n**	**%**	**n**	**%**
INTERVENTION ARRANGEMENTS							
Arranged to stop anticoagulants	No	3/29	10.3%	2/30	6.7%	5/59	8.5%
	Yes	6/29	20.7%	11/30	36.7%	17/59	28.8%
	N/A	20/29	69.0%	17/30	56.7%	37/59	62.7%
PET-CT SCAN							
PET-CT scan performed		1/1	100.0%	28/30	93.3%	29/31	93.5%
If PET scan not performed, reason	Too unwell			2/2	100.0%	2/2	100.0%
If PET scan was performed, results available prior to CT biopsy				28/28	100.0%	28/28	100.0%
Maximum area of uptake biopsied		0/1	0.0%	20/27	74.1%	20/28	71.4%
If maximum area not biopsied, reason	Inaccessible	0/1	0.0%	2/7	28.6%	2/8	25.0%
	Extra-pleural lesion biopsied	0/1	0.0%	1/7	14.3%	1/8	12.5%
	No dominant/localising focus	0/1	0.0%	2/7	28.6%	2/8	25.0%
	Not documented	0/1	0.0%	1/7	14.3%	1/8	12.5%
	Not attempted	1/1	100.0%	1/7	14.3%	2/8	25.0%
CT-GUIDED BIOPSY (PLEURA)							
CT-guided biopsy performed		26/29	89.7%	25/30	83.3%	51/59	86.4%
Number of attempts at taking biopsy	(mean, SD)	2.0	1.4	1.8	1.4	1.9	1.4
If no biopsy, reason	Did not attend	1/3	33.3%	2/5	40.0%	3/8	37.5%
	Too unwell	0/3	0.0%	1/5	20.0%	1/8	12.5%
	Patient died before biopsy	0/3	0.0%	1/5	20.0%	1/8	12.5%
	Insufficient pleural thickening	2/3	66.7%	1/5	20.0%	3/8	37.5%
Laterality (left)		5/26	19.2%	15/25	60.0%	20/51	39.2%
Maximum area of pleural thickening biopsied		19/23	82.6%	18/25	72.0%	37/48	77.1%
Number of passes[1]	(mean, SD)	4	1.7	4	1.5	4	1.6
18g co-axial system used		24/26	92.3%	23/25	92.0%	47/51	92.2%
IMMEDIATE COMPLICATIONS							
Pain at site of biopsy		0/26	0.0%	0/25	0.0%	0/51	0.0%
Pneumothorax		1/26	3.8%	0/25	0.0%	1/51	2.0%
Bruising		0/26	0.0%	0/25	0.0%	0/51	0.0%
Bleeding		0/26	0.0%	0/25	0.0%	0/51	0.0%
Failed biopsy		0/26	0.0%	0/25	0.0%	0/51	0.0%

Notes: *Where denominators do not match total expected numbers, this indicates missing data. CT=computed tomography, PET=positron emission tomography, SD=standard deviation.*

[1] Data missing for 8 patients (3, 5).

Table S6: Sensitivity analysis: a) excluding patients that did not receive the intervention (for outcomes compared by allocation) and b) using a best/worst case simple imputation for missing primary outcome data due to death, withdrawal or loss to follow-up before the intervention took place

a)

**Outcome** [1]	**Randomised to CT guided biopsy only (n=26)**		**Randomised to PET-CT and CT guided biopsy (n=25)**		**Effect**[1]	**p-**
	**median**	**IQR**	**median**	**IQR**	**(95% CI)**	**value**
Correct diagnosis of pleural malignancy (n,%)	21/25	84.0%	19/22	86.4%	RR= 1.02 (0.80,1.31)	0.87
					RD=0.02 (-0.19,0.23)	0.87
Time to diagnosis (days)	35	(20.0, 168.0)	56	(22.0, 375.0)	SHR=0.71 (0.34,1.47)	0.36
Time to death (days)	244	(168, 360)	354	(182, 375)	HR=0.81 (0.29,2.30)	0.70
Total invasive procedures undertaken to confirm diagnosis[2]	1.0	(1.0, 1.0)	1.0	(1.0, 1.0)	IRR=0.94 (0.57,1.55)	0.81
Total hospital attendances undertaken to confirm diagnosis[3]	0.0	(0.0, 1.0)	0.5	(0.0, 2.5)	IRR=1.3 (0.55,3.06)	0.55

Notes: *CT=computed tomography, PET=positron emission tomography, IQR=inter-quartile range, CI=confidence interval.* [1] *RD = risk difference, RR = risk ratio, SHR=sub-hazard ratio, HR=hazard ratio, IRR=incident rate ratio.*
[1] *Time to chemotherapy and procedure-related adverse event outcomes were unchanged by excluding patients who did not receive the intervention,* [2] *Data missing for 1 patient (1,0),* [3] *Data missing for 2 patient (1,1),*

b)

**Outcome**	**Effect (95% CI)**	**p-value**
Correct diagnosis of pleural malignancy (n,%) – worse case imputation – incorrect diagnosis	RR= 1.00 (95% CI: 0.78,1.28)<br>RD=0.00 (95% CI: -0.20,0.20)	>0.99<br>>0.99
Correct diagnosis of pleural malignancy (n,%) – best case imputation – correct diagnosis	RR= 1.03 (95% CI: 0.84,1.28)<br>RD=0.03 (95% CI: -0.15,0.21	0.73<br>0.73

Table S7: Cross tabulations of pleural malignancy diagnosis from second biopsy and final diagnosis at 12 months (all randomised participants, complete case analysis)

**Randomised to CT-guided biopsy only**	**Final diagnosis at 12 months** Malignant (n=19)	Not malignant (n=7)	**Sensitivity** (95% CI)	**Specificity** (95% CI)	**PPV** (95% CI)	**NPV** (95% CI)
**Diagnosis of pleural malignancy from second biopsy**:			0.79 (0.54,0.94)	1.00 (0.59,1.00)	1.00 (0.78,1.00)	0.64 (0.31,0.89)
Yes (n=15)	15	0				
No (n=11)	4	7				

**Randomised to PET-CT and CT-guided biopsy**	**Final diagnosis at 12 months** Malignant (n=16)	Not malignant (n=8)	**Sensitivity** (95% CI)	**Specificity** (95% CI)	**PPV** (95% CI)	**NPV** (95% CI)
**Diagnosis of pleural malignancy from second biopsy**:			0.81 (0.54,0.96)	1.00 (0.63,1.00)	1.00 (0.75,1.0)	0.73 (0.39-0.94)
Yes (n=13)	13	0				
No (n=11)	3	8				

***Note**: CT=computed tomography, PET=positron emission tomography, CI=confidence interval, PPV=positive predictive value, NPV=negative predictive value.*

Table S8: Secondary outcomes: additional detail

		Randomised to CT guided biopsy only (n=29)		Randomised to PET-CT and CT guided biopsy (n=30)		Overall (n=59)	
		n	%	n	%	n	%
NUMBER OF INVASIVE PROCEDURES:							
Total overall	0	2/28	7.1%	3/30	10.0%	5/58	8.6%
	1	23/28	82.1%	24/30	80.0%	47/58	81.0%
	2	3/28	10.7%	3/30	10.0%	6/58	10.3%
Total undertaken to confirm diagnosis	0	2/28	7.1%	3/30	10.0%	5/58	8.6%
	1	24/28	85.7%	25/30	83.3%	49/58	84.5%
	2	2/28	7.1%	2/30	6.7%	4/58	6.9%
NUMBER OF HOSPITAL ATTENDANCES:							
Total overall	0	7/28	25.0%	6/28	21.4%	13/56	23.2%
	1	6/28	21.4%	3/28	10.7%	9/56	16.1%
	2	1/28	3.6%	7/28	25.0%	8/56	14.3%
	3	0/28	0.0%	2/28	7.1%	2/56	3.6%
	4	2/28	7.1%	3/28	10.7%	5/56	8.9%
	5-10	8/28	28.6%	4/28	14.3%	12/56	21.4%
	11-20	3/28	10.7%	3/28	10.7%	6/56	10.7%
	21-30	1/28	3.6%	0/28	0.0%	1/56	1.8%
Total undertaken to confirm diagnosis	0	14/27	51.9%	14/28	50.0%	29/56	51.8%
	1	7/27	25.9%	4/28	14.3%	11/56	19.6%
	2	3/27	11.1%	4/28	14.3%	7/56	12.5%
	3	1/27	3.7%	1/28	3.6%	2/56	3.6%
	4	1/27	3.7%	2/28	7.1%	3/56	5.4%
	5-10	0/27	0.0%	3/28	10.7%	3/56	5.4%
	11-20	1/27	3.7%	0/28	0.0%	1/56	1.8%
PET-CT SCAN:							
Maximum SUV[1, 2]	(median, IQR)			6.3	(3.8, 11.9)		

Notes:
Where denominators do not match total expected numbers, this indicates missing data. CT=computed tomography, PET=positron emission tomography, IQR=inter-quartile range, SUV=standardised uptake value.
[1] Data missing for 6 patients randomised to PET-CT and CT-biopsy
[2] One patient randomised to CT-guided biopsy only also had a PET-CT scan: maximum SUV was 6.9.

Table S9 A-D: Prediction of diagnosis of malignancy by baseline serum mesothelin levels (all randomised patients) and baseline maximum SUV (patients randomised to PET-CT and CT-guided biopsy)

9A: Diagnosis of pleural malignancy

		Diagnosed with pleural malignancy by the end of follow-up: Yes (n=35)	No (n=24)	Sensitivity (95% CI)	Specificity (95% CI)	PPV (95% CI)	NPV (95% CI)	AUC
Baseline serum mesothelin[1]:				0.69 (0.50,0.84)	0.76 (0.53,0.92)	0.82 (0.62,0.94)	0.62 (0.41,0.80)	0.78[2,3,4]
High (>2)	(n=27)	22	5					
Low (≤2)	(n=26)	10	16					

9B: Diagnosis of mesothelioma (sensitivity analysis)

		Diagnosed with mesothelioma by the end of follow-up: Yes (n=32)	No (n=27)	Sensitivity (95% CI)	Specificity (95% CI)	PPV (95% CI)	NPV (95% CI)	AUC
Baseline serum mesothelin[1]:				0.73 (0.54,0.88)	0.78 (0,56,0.93)	0.82 (0.62,0.94)	0.69 (0.48,0.86)	0.83[2,3,5]
High (>2)	(n=27)	22	5					
Low (≤2)	(n=26)	8	18					

9C: Diagnosis of pleural malignancy (using a data defined cut point)

		Diagnosed with pleural malignancy by the end of follow-up: Yes (n=16)	No (n=14)	Sensitivity (95% CI)	Specificity (95% CI)	PPV (95% CI)	NPV (95% CI)	AUC
Maximum SUV[6]:				0.77 (0.46-0.95)	0.91 (0.59-1.0)	0.91 (0.59-1.0)	0.77 (0.46-0.95)	0.92[7]
High (>7.4)[8]	(n=11)	10	1					
Low (≤7.4)	(n=13)	3	10					

9D: Diagnosis of pleural malignancy (using a clinically defined cut point[9])

		Diagnosed with pleural malignancy by the end of follow-up: Yes (n=16)	No (n=14)	Sensitivity (95% CI)	Specificity (95% CI)	PPV (95% CI)	NPV (95% CI)	AUC
Maximum SUV:				0.92 (0.64,1.0)	0.36 (0.11,0.69)	0.63 (0.38,0.84)	0.80 (0.28,1.0)	0.92[10]
High (>3.5)	(n=19)	12	7					
Low (≤3.5)	(n=5)	1	4					

Notes: *CT=computed tomography, PET=positron emission tomography, CI=confidence interval. PPV= positive predictive value, NPV= negative predictive value, AUC=area under ROC (Receiver Operating Characteristic) curve.*

[1] *missing for 6 patients (1 randomised to CT-biopsy only, 5 randomised to PET-CT and CT-biopsy)*

[2] *logistic regression models were adjusted for age, sex, history of respiratory and pleural disease*

[3] *One outlier (that didn't receive the study CT biopsy) was omitted from this regression analysis due to poor model fit*

[4] *sensitivity analysis omitting patients who did not receive the intervention (n=47) gave sensitivity: 0.69 (0.50-0.84); specificity: 0.87 (0.60-0.98); NPV: 0.57 (0.35-0.77); PPV: 0.92 (0.73,0.99); AUC: 0.80*

[5] *sensitivity analysis omitting patients who did not receive the intervention (n=47) gave sensitivity: 0.73 (0.54-0.88); specificity: 0.88 (0.64-0.99); NPV: 0.65 (0.43-0.84); PPV: 0.92 (0.73,0.99); AUC: 0.83*

[6] *missing for 6 patients randomised to PET-CT and CT-biopsy (two patients did not have their PET-CT scan and maximum SUV was not done or not documented for four patients)*

[7] *sensitivity analysis omitting patients who did not receive the intervention (n=22) gave sensitivity: 0.77 (0.46-0.95), specificity: 1.00 (0.66-1.00), NPV: 0.75 (0.43-0.95), PPV 1.00 (0.67,1.00), AUC: 0.92*

[8] *cut point of 7.4 defined using ROC analysis (using 'nearest to top left-hand corner' method)*

[9] *this was an ancillary (post-hoc) analysis*

[10] *sensitivity analysis omitting patients who did not receive the intervention (n=22) gave sensitivity: 0.92 (0.64-1.0); specificity: 0.33 (0.08-0.70); NPV: 0.75 (0.19-0.99); PPV 0.67 (0.41,0.87); AUC: 0.92*

Table S10A: Further follow-up data: general (all randomised patients)

		Randomised to CT guided biopsy only (n=29)		Randomised to PET-CT and CT guided biopsy (n=30)		Overall (n=59)	
		n	%	n	%	n	%
**1-2 WEEK FOLLOW-UP**							
Follow-up visit attended		23/28	82.1%	23/27	85.2%	46/55	83.6%
if no, reason	Patient declined any further follow up	1/5	20%	0/4	0%	1/9	11%
	Patient too unwell	1/5	20%	0/4	0%	1/9	11%
	Patient died	1/5	20%	0/4	0%	1/9	11%
	Other	2/5	40%	4/4	100%	6/9	67%
OTHER DISEASE RELATED CLINICAL VISITS							
District nurse visits		0/25	0.0%	5/23	21.7%	5/48	10.4%
Number of visits	(median, IQR)			1	(1.0, 2.0)	1	(1.0, 2.0)
GP visits		1/25	4.0%	2/23	8.7%	3/48	6.3%
Number of visits	(median, IQR)	1	(1.0, 1.0)	2	(1.0, 3.0)	1	(1.0, 3.0)
Outpatient hospital visits		4/25	16.0%	5/23	21.7%	9/48	18.8%
Number of visits	(median, IQR)	1	(1.0, 1.0)	1	(1.0, 2.0)	1	(1.0, 1.0)
**3 MONTH FOLLOW-UP**							
Follow-up visit attended		21/27	77.8%	20/27	74.1%	41/54	75.9%
if no, reason	Patient too unwell	1/6	17%	0/7	0%	1/13	8%
	Patient died	2/6	33%	3/7	43%	5/13	38%
	Other	3/6	50%	4/7	57%	7/13	54%
PROCEDURES SINCE LAST FOLLOW-UP							
Any procedures since last follow-up		3/24	12.5%	3/27	11.1%	6/51	11.8%
Therapeutic aspiration		1/3	33.3%	1/3	33.3%	2/6	33.3%
laterality (left)		0/1	0%	0/1	0%	0/2	0%
Number of aspirations	(mean, SD)	1		1		1	0.0
Medical thoracoscopy		0/3	0.0%	0/3	0.0%	0/6	0.0%
Surgical thoracoscopy		1/3	33.3%	0/3	0.0%	1/6	16.7%
laterality (left)		0/1	0%			0/1	0%
USS guided biopsy		0/3	0.0%	0/3	0.0%	0/6	0.0%
CT guided biopsy		1/3	33.3%	2/3	66.7%	3/6	50.0%
laterality (left)		0/1	0%	0/2	0%	0/3	0%
TREATMENT/SUPPORT SINCE LAST FOLLOW-UP							
Any treatment/support since last follow-up		6/25	24.0%	7/27	25.9%	13/52	25.0%
First line chemotherapy		2/6	33.3%	4/7	57.1%	6/13	46.2%
Second line chemotherapy		0/6	0.0%	0/7	0.0%	0/13	0.0%
Radiotherapy		1/6	16.7%	1/7	14.3%	2/13	15.4%
Other trial involvement		1/6	16.7%	0/7	0.0%	1/13	7.7%
further detail	LUME-meso trial	1/1	100.0%			1/1	100.0%
Palliative care		2/6	33.3%	4/7	57.1%	6/13	46.2%
Cordotomy		0/6	0.0%	0/7	0.0%	0/13	0.0%
Repeated treatment		0/6	0.0%	0/7	0.0%	0/13	0.0%
OTHER DISEASE RELATED CLINICAL VISITS							
District nurse visits		3/24	12.5%	2/22	9.1%	5/46	10.9%
Number of visits	(median, IQR)	30	(15.0, 40.0)	20	(4.0, 36.0)	30	(15.0, 36.0)
GP visits		9/24	37.5%	6/22	27.3%	15/46	32.6%
Number of visits	(median, IQR)	1	(1.0, 3.0)	1	(1.0, 2.0)	1	(1.0, 3.0)
Outpatient hospital visits		15/24	62.5%	11/23	47.8%	26/47	55.3%

		Randomised to CT guided biopsy only (n=29)		Randomised to PET-CT and CT guided biopsy (n=30)		Overall (n=59)	
		n	%	n	%	n	%
Number of visits	(median, IQR)	3	(1.0, 4.0)	2	(1.0, 3.0)	3	(1.0, 4.0)
**6 MONTH FOLLOW-UP**							
Follow-up visit attended		19/24	79.2%	21/24	87.5%	40/48	83.3%
if no, reason	Patient declined any further follow up	1/5	20%	0/2	0%	1/7	14%
	Patient too unwell	1/5	20%	0/2	0%	1/7	14%
	Patient died	2/5	40%	1/2	50%	3/7	43%
	Other	1/5	20%	1/2	50%	2/7	29%
PROCEDURES SINCE LAST FOLLOW-UP							
Any procedures since last follow-up		4/24	16.7%	1/23	4.3%	5/47	10.6%
Therapeutic aspiration		3/4	75.0%	0/1	0.0%	3/5	60.0%
laterality (left)		0/3	0%			0/3	0%
Number of aspirations[1]	(mean, SD)	1	0.0			1	0.0
Medical thoracoscopy		0/4	0.0%	0/1	0.0%	0/5	0.0%
Surgical thoracoscopy		1/4	25.0%	0/1	0.0%	1/5	20.0%
laterality (left)		0/1	0%			0/1	0%
USS guided biopsy		0/4	0.0%	0/1	0.0%	0/5	0.0%
CT guided biopsy		1/4	25.0%	1/1	100.0%	2/5	40.0%
laterality (left)		1/1	100%	1/1	100%	2/2	100%
TREATMENT/SUPPORT SINCE LAST FOLLOW-UP							
Any treatment/support since last follow-up		6/24	25.0%	3/24	12.5%	9/48	18.8%
First line chemotherapy		3/6	50.0%	2/3	66.7%	5/9	55.6%
Second line chemotherapy		0/6	0.0%	0/3	0.0%	0/9	0.0%
Radiotherapy		1/6	16.7%	1/3	33.3%	2/9	22.2%
Other trial involvement		2/6	33.3%	0/3	0.0%	2/9	22.2%
further detail	MARS2 trial	1/2	50.0%			1/2	50.0%
	MESO TRAP trial	1/2	50.0%			1/2	50.0%
Palliative care		1/6	16.7%	2/3	66.7%	3/9	33.3%
Cordotomy		0/6	0.0%	0/3	0.0%	0/9	0.0%
Repeated treatment type		0/6	0.0%	0/3	0.0%	0/9	0.0%
OTHER DISEASE RELATED CLINICAL VISITS							
District nurse visits		2/21	9.5%	3/22	13.6%	5/43	11.6%
Number of visits[2]	(median, IQR)	10	(10.0, 10.0)	21	(8.0, 23.0)	16	(9.0, 22.0)
GP visits		6/21	28.6%	9/22	40.9%	15/43	34.9%
Number of visits	(median, IQR)	2	(1.0, 2.0)	1	(1.0, 2.0)	1	(1.0, 2.0)
Outpatient hospital visits		15/22	68.2%	14/22	63.6%	29/44	65.9%
Number of visits[3]	(median, IQR)	3	(1.0, 4.0)	1	(0.0, 4.0)	2	(1.0, 4.0)
**12 MONTH FOLLOW-UP**							
Follow-up visit attended		10/19	52.6%	14/18	77.8%	24/37	64.9%
if no, reason	Patient too unwell	3/9	33%	1/4	25%	4/13	31%
	Patient died	4/9	44%	2/4	50%	6/13	46%
	Other	2/9	22%	1/4	25%	3/13	23%
PROCEDURES SINCE LAST FOLLOW-UP							
Any procedures since last follow-up		0/19	0.0%	2/18	11.1%	2/37	5.4%
Therapeutic aspiration				0/2	0.0%	0/2	0.0%
Medical thoracoscopy				0/2	0.0%	0/2	0.0%

		Randomised to CT guided biopsy only (n=29)		Randomised to PET-CT and CT guided biopsy (n=30)		Overall (n=59)	
		**n**	**%**	**n**	**%**	**n**	**%**
Surgical thoracoscopy				0/2	0.0%	0/2	0.0%
USS guided biopsy				1/2	50.0%	1/2	50.0%
laterality (left)				1/1	100%	1/1	100%
CT guided biopsy				1/2	50.0%	1/2	50.0%
laterality (left)				1/1	100%	1/1	100%
TREATMENT/SUPPORT SINCE LAST STUDY FOLLOW-UP							
Any treatment/support since last follow-up		7/19	36.8%	5/18	27.8%	12/37	32.4%
First line chemotherapy		1/7	14.3%	3/5	60.0%	4/12	33.3%
Second line chemotherapy		1/7	14.3%	0/5	0.0%	1/12	8.3%
Radiotherapy		1/7	14.3%	0/5	0.0%	1/12	8.3%
Other trial involvement		1/7	14.3%	0/5	0.0%	1/12	8.3%
further detail	Zol-A trial	1/1	100.0%			1/1	100.0%
Palliative care		4/7	57.1%	3/5	60.0%	7/12	58.3%
Cordotomy		0/7	0.0%	0/5	0.0%	0/12	0.0%
Repeated treatment		0/7	0.0%	0/5	0.0%	0/12	0.0%
OTHER DISEASE RELATED CLINICAL VISITS							
District nurse visits		2/16	12.5%	2/14	14.3%	4/30	13.3%
Number of visits[4]	(median, IQR)	11	(11.0, 11.0)	2	(1.0, 3.0)	3	(1.0, 11.0)
GP visits		9/16	56.3%	6/14	42.9%	15/30	50.0%
Number of visits	(median, IQR)	1	(1.0, 2.0)	2	(1.0, 2.0)	1	(1.0, 2.0)
Outpatient hospital visits		11/17	64.7%	10/15	66.7%	21/32	65.6%
Number of visits	(median, IQR)	5	(1.0, 6.0)	3	(2.0, 4.0)	3	(2.0, 5.0)

Notes: *Where denominators do not match total expected numbers, this indicates missing data. CT=computed tomography, PET=positron emission tomography, IQR=inter-quartile range, SD=standard deviation, USS=ultrasound, GP=general practitioner.*

[1] *Data missing for 1 patient (1,0),* [2] *Data missing for 1 patient (1,0),* [3] *Data missing for 3 patients (1,2),* [4] *Data missing for 1 patient (1,0)*

Table S10B: Further follow-up data: chemotherapy and radiotherapy in patients with a malignant diagnosis

		Randomised to CT guided biopsy only (n=19)		Randomised to PET-CT and CT guided biopsy (n=16)		Overall (n=35)	
		n	%	n	%	n	%
CHEMOTHERAPY							
Instances of chemotherapy following a malignant diagnosis[1]	Events/patients, %	6/5	26.3%	6/5	33.3%	12/10	29.4%
FIRST LINE CHEMOTHERAPY							
Instances of first-line chemotherapy [2]	Events/patients, %	5/5	26.3%	6/5	33.3%	11/10	29.4%
First-line chemotherapy agent round 1	Erlotinib	0/5	0.0%	1/5	20.0%	1/10	10.0%
	Carboplatin & pemetrexed	3/5	60.0%	1/5	20.0%	4/10	40.0%
	Cisplatin & pemetrexed	2/5	40.0%	3/5	60.0%	5/10	50.0%
Duration of chemotherapy (days)	Median (IQR)	100	(96.0, 111.0)	85	(84.0, 91.0)	94	(85.0, 111.0)
Number of cycles	Median (IQR)	5	(5.0, 6.0)	4	(3.0, 5.0)	5	(4.0, 6.0)
First-line chemotherapy agent round 2	Carboplatin & pemetrexed			1/1	100.0%	1/1	100.0%
Duration of chemotherapy (days)	Median (IQR)			120	(120, 120)	120	(120, 120)
Number of cycles	Median (IQR)			3	(3.0, 3.0)	3	(3.0, 3.0)
SECOND LINE CHEMOTHERAPY							
Instances of second-line chemotherapy [3]	Events/patients, %	1/1	5.3%	0/0	0.0%	1/1	2.9%
Second-line chemotherapy agent	Vinorelbine	1/1	100.0%			1/1	100.0%
Duration of chemotherapy (days)	Median (IQR)	92	(92.0, 92.0)			92	(92.0, 92.0)
Number of cycles	Median (IQR)	6	(6.0, 6.0)			6	(6.0, 6.0)
RADIOTHERAPY							
Instances of radiotherapy following a malignant diagnosis[4]	events/patients, %	3/3	15.8%	3/2	13.3%	6/5	14.7%
Procedure tract site radiotherapy		2/3	66.7%	0/2	0.0%	2/5	40.0%
Duration of radiotherapy (days)	Mean (SD)	2	0.7			2	0.7
Number of fractions	Mean (SD)	3	1			3	1
Chest wall invasion		0/3	0.0%	0/2	0.0%	0/5	0.0%
Pain (round 1)		1/3	33.3%	2/2	100.0%	3/5	60.0%
Duration of radiotherapy (days)	Mean (SD)	0		0	0.0	0	0.0
Number of fractions	Mean (SD)	1		3	3	2	2
Pain (round 2)				1/1	100.0%	1/1	100.0%

		Randomised to CT guided biopsy only (n=19)		Randomised to PET-CT and CT guided biopsy (n=16)		Overall (n=35)	
		**n**	**%**	**n**	**%**	**n**	**%**
Duration of radiotherapy (days)	Mean (SD)			0		0	.
Number of fractions	Mean (SD)			5		5	.

Notes: *CT=computed tomography, PET=positron emission tomography, IQR=inter-quartile range, SD=standard deviation.*
[1] Data missing for 1 patient (0,1), [2] Data missing for 1 patient (0,1) , [3] Data missing for 1 patient (0,1) , [4] Data missing for 1 patient (0,1) .

Table S11: Independent review of histology and impact on diagnostic pathway from PET

	Randomised to CT guided biopsy only (n=29)		Randomised to PET-CT and CT guided biopsy (n=30)		Effect size	p-
	n	%	n	%	(95% CI)	value
Initial diagnosis from independent histology review[1]						
Malignant	14/26	53.8%	12/25	48.0%		
Benign	6/26	23.1%	9/25	36.0%		
Suspicious or non-diagnostic biopsy	6/26	23.1%	4/25	16.0%		
PET-CT scan impact:-						
- in patients with a correct diagnosis from the second biopsy						
no site change or upstaging	1/1	100.0%	7/21	33.3%		
upstaging only	0/1	0.0%	2/21	9.5%		
site change only 2,3	0/1	0.0%	8/21	38.1%		
site change and upstaging	0/1	0.0%	4/21	19.0%		
- in patients without a correct diagnosis from the second biopsy						
no site change or upstaging			2/3	66.7%		
site change only			1/3	33.3%		

Notes: *Where denominators do not match total expected numbers, this indicates missing data. CT=computed tomography, PET=positron emission tomography, CI=confidence interval, RR=relative risk, RD=Risk difference*
[1]no additional malignant diagnoses were identified from the independent review, [2] 2 were correct non-malignant diagnoses, [3] One patient was missing upstaging data.

Table S12: Longitudinal secondary outcome: serum mesothelin (all randomised patients)

		Randomised to CT guided biopsy only (n=29)		Randomised to PET-CT and CT guided biopsy (n=30)		Overall (n=59)	
		**median**	**IQR**	**median**	**IQR**	**median**	**IQR**
Serum mesothelin (nmol/L)	Baseline[1]	2.5	(1.6, 4.3)	1.9	(1.3, 2.6)	2.2	(1.4, 3.5)
	6 months[2]	2.1	(1.3, 3.2)	1.1	(1.0, 1.7)	1.7	(1.1, 2.6)
	12 months[3]	4.5	(1.5, 5.3)	1.3	(1.0, 1.7)	1.6	(1.3, 4.4)
Serum mesothelin in patients with a positive mesothelioma diagnosis	Baseline[4]	2.9	(1.9, 5.0)	2.7	(2.2, 4.5)	2.9	(2.1, 4.5)
	6 months[5]	2.6	(2.0, 3.5)	1.4	(1.1, 2.7)	2.1	(1.7, 3.5)
	12 months[6]	5.3	(5.0, 7.1)	1.6	(1.5, 1.7)	5.0	(1.7, 5.3)
Serum mesothelin in patients without a positive mesothelioma diagnosis	Baseline[7]	1.7	(1.4, 2.4)	1.4	(1.0, 1.8)	1.4	(1.2, 2.0)
	6 months[8]	1.5	(0.9, 2.1)	1.0	(0.8, 1.1)	1.1	(0.9, 1.9)
	12 months[9]	1.5	(1.4, 3.9)	1.1	(0.9, 1.5)	1.4	(1.0, 1.8)
Reason for missing baseline mesothelin result (n,%)	Sample sent but result not available	1/29	3%	5/30	17%	6/59	10%
	Mesothelin level out of assay range	0/29	0%	1/30	3%	1/59	2%
	N/A (result not missing)	28/29	97%	24/30	80%	52/59	88%
Reason for missing 6 month mesothelin result (n,%)	Sample sent but result not available	1/29	3%	1/30	3%	2/59	3%
	Follow-up visit not attended in person (e.g. due to patient death or phone follow-up)	11/29	38%	17/30	57%	28/59	47%
	Other	2/29	7%	2/30	7%	4/59	7%
	N/A (result not missing)	15/29	52%	10/30	33%	25/59	42%
Reason for missing 12 month mesothelin result (n,%)	Follow-up visit not attended in person	19/29	66%	21/30	70%	40/59	68%
	Mesothelin level out of assay range	1/29	3%	0/30	0%	1/59	2%
	Other	3/29	10%	3/30	10%	6/59	10%
	N/A (result not missing)	6/29	21%	6/30	20%	12/59	20%

Notes: *CT=computed tomography , PET=positron emission tomography, IQR=inter-quartile range.*
[1] Data missing for 7 patients (1,6), [2] Data missing for 34 patients (14,20), [3] Data missing for 47 patients (23,24), [4] Data missing for 3 patients (0,3), [5] Data missing for 19 patients (10,9), [6] Data missing for 27 patients (16,11), [7] Data missing for 4 patients (1,3), [8] Data missing for 15 patients (4,11), [9] Data missing for 20 patients (7,13).

Table S13 Adverse events during follow-up (all randomised patients)

Event	Randomised to CT guided biopsy only (n=29)				Randomised to PET-CT and CT guided biopsy (n=30)			
	AE		SAE[1]		AE		SAE	
	Events/ patients	%	Events/ patients	%	Events/ patients	%	Events/ patients	%
RELATED TO CT-BIOPSY (trial biopsy or subsequent biopsy)								
Pain at site of biopsy[2]	3/3	12.5%	0/0	0.0%	3/3	12.5%	1/1	33.3%
Bleeding and bruising	0/0	0.0%			1/1	4.2%	1/1	100.0%
Pneumothorax[3]	1/1	4.2%	0/0	0.0%	0/0	0.0%		
RELATED TO TREATMENTS								
Chemotherapy-related complications	5/3	11.1%	1/1	33.3%	3/2	8.3%	1/1	50.0%
Pneumonia	1/1	3.7%	1/1	100.0%	0/0	0.0%		
Other infection	4/3	11.1%	3/2	66.7%	1/1	4.2%	0/0	0.0%
RELATED TO DISEASE PROGRESSION								
Admission to hospice with disease progression	1/1	3.6%	1/1	3.6%	1/1	3.7%	1/1	3.7%
ED attendance for chest pain[4]	1/1	3.6%	1/1	100.0%	4/3	11.1%	2/2	66.7%
ED attendance for breathlessness[5]	6/5	17.9%	3/2	40.0%	4/4	15.4%	2/2	50.0%
ED attendance for other general deterioration	6/6	21.4%	4/4	66.7%	5/4	15.4%	2/2	50.0%
OTHER ADVERSE EVENTS (SERIOUS EVENTS ONLY)								
Other non-fatal serious adverse events	9/8	28.6%	9/8	28.6%	5/4	13.8%	5/4	13.8%
Deaths	9/9	31.0%	9/9	31.0%	9/9	30.0%	9/9	30.0%
TOTAL RECOREDED ADVERSE EVENTS:	46/19	65.5%	32/15	51.7%	36/17	56.7%	24/14	46.7%

Notes: *CT=computerised tomography, PET=positron emission tomography, AE=adverse event, SAE=serious adverse event, ED=emergency department. [1] the criteria for a serious adverse event are: complications that were life-threatening or caused hospitalisation, increased length of hospital admission, persistent or significant disability or death, [2] this patient had a PET-CT scan but did not receive the study CT-biopsy (PET-CT arm), [3] this is the same patient with pneumothorax reported as an immediate complication of the intervention – see Table S5), [4] one event was in a patient in the PET-CT arm that had a PET-CT scan but did not receive the study CT-biopsy: the event was classified as an SAE, [5] one event was in a patient in the CT-only arm that did not receive the study CT-biopsy. The event was not classified as an SAE.*

Supplementary References

1. Lambert, P.C., *The Estimation and Modeling of Cause-specific Cumulative Incidence Functions Using Time-dependent Weights*. Stata J, 2017. **17** (1): p181-207.
