# Supplementary material for: PET-CT-guided versus CT-guided biopsy in suspected malignant pleural thickening: a randomised trial
Source: Eur Respir J. 2024 Feb 1;63(2):2301295. doi: 10.1183/13993003.01295-2023 (PMC10831139; doi:10.1183/13993003.01295-2023)
Supplement: Supplementary file 2 [file ERJ-01295-2023.Shareable.pdf]

SHAREABLE PDF

PET-CT-guided *versus* CT-guided biopsy in suspected malignant pleural thickening: a randomised trial

Duneesha de Fonseka, David T. Arnold, Helena J.M. Smartt, Lucy Culliford, Louise Stadon, Emma Tucker, Anna Morley, Natalie Zahan-Evans, Anna C. Bibby, Geraldine Lynch, Eleanor Mishra, Shahul Khan, Mohammed Haris, Henry Steer, Leon Lewis, Alina Ionescu, John Harvey, Kevin Blyth, Najib M. Rahman, Anthony E. Edey, Chris A. Rogers and Nick A. Maskell

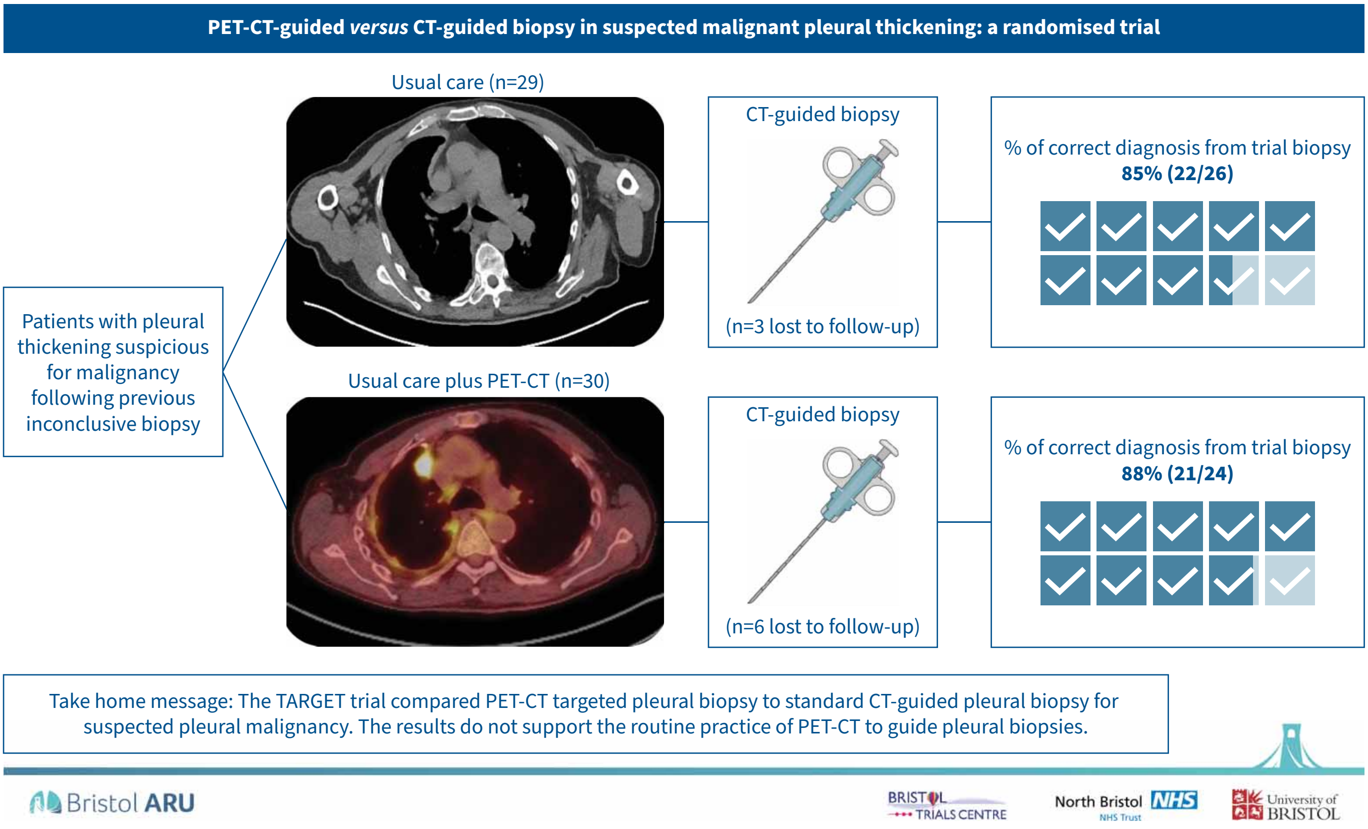

GRAPHICAL ABSTRACT Summary of the TARGET trial. PET: positron emission tomography; CT: computed tomography.

EUROPEAN RESPIRATORY JOURNAL
ORIGINAL RESEARCH ARTICLE
D. DE FONSEKA ET AL.

SHAREABLE PDF

PET-CT-guided *versus* CT-guided biopsy in suspected malignant pleural thickening: a randomised trial

Duneesha de Fonseka[1], David T. Arnold[2,3], Helena J.M. Smartt[4], Lucy Culliford[4], Louise Stadon[2,3], Emma Tucker[2,3], Anna Morley[2,3], Natalie Zahan-Evans[5], Anna C. Bibby[2,3], Geraldine Lynch[2,3], Eleanor Mishra[6,7], Shahul Khan[8], Mohammed Haris[8], Henry Steer[9], Leon Lewis[1], Alina Ionescu[10], John Harvey[2,3], Kevin Blyth[11], Najib M. Rahman[12,13,14], Anthony E. Edey[3], Chris A. Rogers[4] and Nick A. Maskell[2,3]

[1]Academic Directorate of Respiratory Medicine, University of Sheffield, Sheffield, UK. [2]Academic Respiratory Unit, University of Bristol, Southmead Hospital, Bristol, UK. [3]North Bristol NHS Trust, Bristol, UK. [4]Bristol Trials Centre, Medical School, University of Bristol, Bristol, UK. [5]Royal Cornwall Hospital NHS Trust, Truro, UK. [6]University of East Anglia, Norwich, UK. [7]Norfolk and Norwich University Hospitals NHS Foundation Trust, Norwich, UK. [8]Royal Stoke University Hospital, Stoke, UK. [9]Gloucestershire Hospitals NHS Trust, Gloucester, UK. [10]Aneurin Bevan University Hospital Trust, Newport, UK. [11]University of Glasgow, Glasgow, UK. [12]Oxford NIHR Biomedical Research Centre, Oxford, UK. [13]Nuffield Department of Medicine, University of Oxford, Oxford, UK. [14]Chinese Academy of Medical Sciences Oxford Institute, Oxford, UK.

Corresponding author: David T. Arnold (arnold.dta@gmail.com)

Check for updates

Shareable abstract (@ERSpublications)
The TARGET trial was a multicentre randomised trial comparing PET-CT targeted pleural biopsy to standard CT-guided pleural biopsy for suspected pleural malignancy. The results do not support the practice of PET-CT to guide pleural biopsies. https://bit.ly/40HOY6p

Cite this article as: de Fonseka D, Arnold DT, Smartt HJM, *et al*. PET-CT-guided *versus* CT-guided biopsy in suspected malignant pleural thickening: a randomised trial. *Eur Respir J* 2024; 63: 2301295 [DOI: 10.1183/13993003.01295-2023].

This extracted version can be shared freely online.

Copyright ©The authors 2024.

This version is distributed under the terms of the Creative Commons Attribution Licence 4.0.

This article has an editorial commentary: https://doi.org/10.1183/13993003.00037-2024

Received: 1 Aug 2023
Accepted: 13 Nov 2023

Abstract

Background Pleural biopsy is the gold standard for diagnosis of pleural malignancy but a significant proportion will have an inconclusive biopsy despite ongoing clinical suspicion of malignancy. We investigated whether positron emission tomography-computed tomography (PET-CT) targeted pleural biopsy is superior to standard CT-guided pleural biopsy following an initial non-diagnostic biopsy.

Methods The TARGET trial was a multicentre, parallel group randomised trial. Patients with a previous inconclusive pleural biopsy but an ongoing suspicion of pleural malignancy were randomised (1:1) to receive either CT-guided biopsy (standard care) or PET-CT followed by a targeted CT biopsy (intervention). The primary outcome was pleural malignancy correctly identified from the trial biopsy.

Results Between September 2015 and September 2018, 59 participants were randomised from eight UK hospital sites: 29 to CT-only followed by targeted biopsy and 30 to PET-CT followed by targeted biopsy. The proportion of pleural malignancy correctly identified was similar between the groups (risk ratio 1.03 (95% CI 0.83–1.29); p=0.77). The sensitivity of the trial biopsy to identify pleural malignancy was 79% (95% CI 54–94%) in the CT-only group *versus* 81% (95% CI 54–96%) in the PET-CT group.

Conclusions The results do not support the practice of PET-CT to guide pleural biopsies in patients with a previous non-diagnostic biopsy. The diagnostic sensitivity in the CT-only group was higher than anticipated and supports the practice of repeating a CT-guided biopsy following an inconclusive result if clinical suspicion of malignancy persists.
